# GRKs as Key Modulators of Opioid Receptor Function

**DOI:** 10.3390/cells9112400

**Published:** 2020-11-02

**Authors:** Laura Lemel, J Robert Lane, Meritxell Canals

**Affiliations:** 1 Division of Physiology, Pharmacology and Neuroscience, School of Life Sciences, Queen’s Medical Centre, University of Nottingham, Nottingham NG7 2UH, UK; laura.lemel@nottingham.ac.uk (L.L.); rob.lane@nottingham.ac.uk (J.R.L.); 2 Centre of Membrane Proteins and Receptors, University of Birmingham and University of Nottingham, Midlands NG7 2UH, UK

**Keywords:** opioid, GPCR, GRK, kinases

## Abstract

Understanding the link between agonist-induced phosphorylation of the mu-opioid receptor (MOR) and the associated physiological effects is critical for the development of novel analgesic drugs and is particularly important for understanding the mechanisms responsible for opioid-induced tolerance and addiction. The family of G protein receptor kinases (GRKs) play a pivotal role in such processes, mediating phosphorylation of residues at the C-tail of opioid receptors. Numerous strategies, such as phosphosite specific antibodies and mass spectrometry have allowed the detection of phosphorylated residues and the use of mutant knock-in mice have shed light on the role of GRK regulation in opioid receptor physiology. Here we review our current understanding on the role of GRKs in the actions of opioid receptors, with a particular focus on the MOR, the target of most commonly used opioid analgesics such as morphine or fentanyl.

## 1. Introduction

Our understanding of the function and regulation of opioid receptors (ORs) has improved dramatically since their identification in the seventies and subsequent cloning in the early 90 s. The first OR to be cloned was the δ opioid receptor (DOR) in 1992 [1,2] followed shortly by the μ (MOR) and the κ (KOR) opioid receptors [3,4,5]. A fourth opioid receptor, that showed high homology to the recently identified MOR, DOR and KOR, was cloned in 1994 [6] but it failed to bind known endogenous and exogenous opioids. This led scientists to name it the opioid receptor-like receptor but is now named the nociceptin receptor (NOR) [7,8]. Its endogenous ligand was later identified by two different groups and named orphanin FQ/nociceptin [8,9]. ORs belong to the superfamily of G protein-coupled receptors (GPCRs). They play crucial roles in the central nervous system (CNS) by regulating the perception of pain, the feeling of well-being, reward and pleasure. Together, the four ORs, mu, delta, kappa, and nociceptin, are privileged targets for the treatment of pain but also neurological and psychiatric disorders [10]. Besides, ORs also play important roles in immune system function, the control of respiration and gastrointestinal function [11,12].

Although opium has been used for millennia, morphine, the primary alkaloid of opium, was only isolated two centuries ago, and opioids such as morphine, oxycodone and fentanyl still remain the mainstay analgesics for the treatment of acute and severe pain. However, due to the expression of ORs in different regions of the nervous system, these opioids also induce several direct and acute side effects including respiratory depression, miosis (or pinpoint pupils), euphoria, drowsiness and constipation. Long-term side effects include tolerance, dependence and addiction. Altogether, these side effects severely limit the use of opioids in the clinic and have contributed, at least in part, to the current opioid epidemic [13]. The physiological actions of current opioid analgesics are mainly mediated by the MOR subtype [14] and molecular changes at the MOR are known to contribute to the chronic effects of opioids, including changes in receptor-effector coupling and in receptor internalization, both of which are tightly controlled by receptor phosphorylation. In this context, G protein receptor kinases (GRKs) are important modulators of opioid receptor function. Here we summarize our current understanding of the actions of GRKs on ORs, with a particular focus on the MOR and directing the reader to relevant literature for existing information on the other OR subtypes.

## 2. Opioid Receptors

To date, the high-resolution structures of the four ORs have been described [15,16,17,18,19,20,21,22,23,24]. Structures in both inactive and active conformations have provided important information for the understanding of the activation mechanisms of these receptors, aided the design of novel drugs using computational approaches [25,26,27] and provided unprecedented insight into ligand selectivity between the different members of the OR family [16]. In addition, the use of conformationally selective nanobodies or miniaturised G proteins as strategies to obtain stable active conformations of ORs have also fuelled significant advances to further understand OR pharmacology in vitro [28,29,30,31].

Opioid receptors are expressed in multiple regions of the brain that contribute to pain perception and are involved in the ascending and the descending nerve pathways that deliver nociceptive information [10]. In the ascending pathway, information starts in primary sensory neurons with nociceptive receptors. It is then transferred to secondary neurons in the spinal cord to the hypothalamus. Finally, information reaches tertiary neurons and travels to the cerebral cortex. The ascending pathway allows for the understanding and feeling of pain. At this stage, endogenous opioid-releasing interneurons in the spinal cord synapse, release endogenous opioid peptides that bind to ORs on sensory neurons, preventing the release of neurotransmitters from primary afferent nociceptors (presynaptic) and causing hyperpolarisation in the postsynaptic neurons. This hyperpolarisation impedes the propagation of the action potential and stops the pain transmission. In brainstem synapses, opioids bind to presynaptic ORs that cause the inhibition of GABA release and activate the descending pathway of pain.

At a cellular level, upon binding of opioid agonists, ORs act via activation of the G_i/o_ G protein family. The Gα_i/o_ subunit released by activation of ORs causes the inhibition of cyclic adenosine monophosphate (cAMP) production by inhibiting the adenylyl cyclase (AC). Therefore, cAMP can no longer activate the cAMP-dependent protein kinase (PKA) responsible for phosphorylating downstream proteins and channels. Presynaptically, the released G_βγ_ subunits bind to and inhibit voltage-gated calcium channels (VGCC), preventing the release of neurotransmitters. Postsynaptically, G_βγ_ subunits activate G protein-coupled inward-rectifying potassium (GIRK) channels, releasing K^+^ from the cell and preventing depolarisation [32]. Other important downstream signaling cascades activated by ORs include the mitogen-activated protein kinase (MAPK) pathways (e.g., ERK, JNK and p38) although the mechanisms underlying these signals are highly ligand- and subtype-dependent [33].

As with most GPCRs, acute MOR signaling is controlled by the phosphorylation of intracellular serine and threonine residues by GRKs and other second messenger kinases [34]. Receptor phosphorylation stabilizes the binding of β-arrestins to the receptor leading to desensitization (G protein uncoupling), formation of endocytic complexes and GPCR internalization. The MOR is a prototypical GPCR for which ligand-dependent phosphorylation “barcodes” have been described which impact β-arrestin recruitment, desensitization and receptor trafficking (see below). Moreover, phosphorylation of MORs has been shown to occur pre- and post-synaptically (albeit with different consequences) [35] and is likely to contribute as a first step to the development of tolerance [36,37]. The sections below summarize the current knowledge on the molecular determinants and mechanisms of OR phosphorylation by GRKs, from studies in purified receptors and model cellular systems to research in tissues and whole animal models (Table 1).

## 3. The Phosphorylation Barcodes of ORs

After prolonged activation by an agonist, most GPCRs present at the plasma membrane undergo phosphorylation by GRKs at specific serine and threonine residues located on intracellular loops or the C-terminus of the receptor. Those specific sites have been identified for many GPCRs and represent a distinct barcode recognized and phosphorylated by specific kinases [34,50]. Phosphorylation by GRKs and subsequent recruitment of β-arrestins are critical regulators of OR signaling and trafficking. Recruitment of GRKs to MOR and subsequent phosphorylation are very rapid [29,42] and precede arrestin binding and desensitization, which reach steady state in several minutes [40]. Importantly, all these processes; phosphorylation, arrestin recruitment and desensitization, are not only agonist-dependent but also dependent on the cell type and the region of the brain in which the receptor and its effectors are expressed [36].

**Figure 1:**
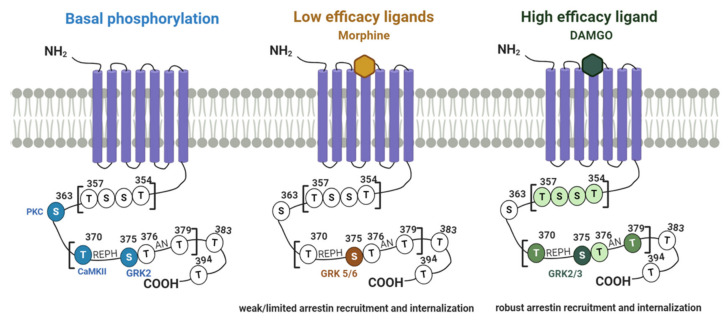
The MOR phosphorylation barcode. MOR residues and kinases involved in constitutive phosphorylation (basal) or upon low and high efficacy agonist-induced phosphorylation (e.g., morphine and DAMGO).

There are 11 potential phosphorylation sites at the C-tail of MOR (Figure 1). Although there are additional Ser/Thr in the intracellular loops (ICLs), it is not clear whether these sites are phosphorylated in a ligand-dependent manner. Eight of the C-tail Ser and Thr residues are in two specific cassettes, ^354^TSST^357^ and ^370^TREHPSTANT^379^ [51], while Ser^363^, Thr^383^ and Thr^394^ are in between these motifs or at the distal region of the C-tail [36,52]. These sites have been identified using site-directed mutagenesis, mass spectrometry and phospho-site-specific antibodies. Once identified, their role in receptor desensitization, β-arrestin recruitment and receptor trafficking has been assessed using electrophysiology, imaging and biochemical and biophysical approaches. Finally, recent studies in transgenic mice have shed light into the physiological impact of such phosphorylation on opioid receptor actions.

Early in vitro studies suggested that similar to MOR internalization, MOR phosphorylation is highly dependent on the opioid agonist bound to the receptor. Zhang et al., (1998) showed that while morphine was unable to induce MOR phosphorylation at endogenous GRK2 levels in HEK293 cells, upon overexpression of GRK2, morphine gained the capacity to induce MOR phosphorylation and internalization [53]. Subsequent experiments demonstrated that Ser^375^ is a key residue that plays a crucial role in ligand-dependent phosphorylation and internalization of MOR [52,54]. Importantly, while all opioid agonists can induce Ser^375^ phosphorylation, the kinase mediating this modification, the ensuing phosphorylation events and subsequent β-arrestin recruitment and receptor endocytosis, are highly dependent on the intrinsic efficacy of the agonist itself [51]. Morphine, a partial agonist at the MOR, induces robust phosphorylation of Ser^375^, while adjacent residues are weakly (Thr^370^, Thr^376^) or not phosphorylated (^354^TSST^357^) [40,42,51,55]. In contrast, full agonists such as Met-enkephalin or its stable analogue peptide [D-Ala2, N-MePhe4, Gly5-ol]-enkephalin (DAMGO), induce sequential and hierarchical phosphorylation of the C-tail of MOR [55]. According to this, phosphorylation of Ser^375^ is the first essential step and immediately followed by phosphorylation of the flanking residues Thr^370^ and Thr^379^ of the ^370^TREHPSTANT^379^ motif, while phosphorylation of Thr^376^ (immediately adjacent to Ser^375^) and the ^354^TSST^357^ motif occur at a slower rate. Mutation of Ser^375^ to Ala strongly reduces phosphorylation of all the other residues, demonstrating its crucial role in the generation of the phosphorylation barcode [55]. Such differences in the phosphorylation profiles have also been demonstrated for other opioid agonists with different efficacies, including buprenorphine, pethidine, nortilidine, oxycodone, oliceridine, methadone, etiatiozene, etorphine, sulfentanyl and fentanyl. Constitutive, ligand-independent, phosphorylation has been described for Ser^363^ as well as for Thr^370^ and Ser^375^ in the ^370^TREHPSTANT^379^ motif. While the physiological relevance of such basal phosphorylation is still unclear, the kinases responsible for this have been proposed to be GRK2 for Ser^375^, Protein kinase C (PKC) for Ser^363^ and CaMKII for Thr^370^ [56]. Finally, although the phosphorylation of two additional residues in the distal region of the C-tail of MOR, Thr^383^ and Thr^394^, has been predicted, it has not been directly observed [51,52]. Understanding the role of these two distal sites represents a challenge for the future as mutations on their own has very little effect but when they are mutated in addition to the residues mentioned above, they potentiate receptor internalization [42,57].

It is now clear that the distinct phosphorylation barcode triggered by opioid agonists is a consequence of the action of different kinases. In an elegant study using siRNA to inhibit the expression of GRK2, 3, 5 and 6, Doll et al., demonstrated that while the phosphorylation induced by DAMGO in HEK293 cells is GRK2- and GRK3-dependent, phosphorylation of the Ser^375^ upon morphine activation is mostly mediated by GRK5 [58]. The action of GRK5 on morphine-activated MOR was confirmed in brain tissue and, in line with previous reports, GRK5-mediated phosphorylation of Ser^375^ was not sufficient to drive multi-site phosphorylation and receptor internalization.

Multiple studies using pharmacological and genetic inhibition, have confirmed the key role of GRK2 in MOR-induced phosphorylation by high efficacy agonists such as Met-enk, DAMGO or fentanyl [40,55]. However, it is also now clear that upon overexpression of GRK2, even low efficacy ligands, such as morphine, are able to induce multi-site phosphorylation of the MOR and, consequently, recruit β-arrestins and internalize the receptor [31,40,53,55,58]. This illustrates the importance of cell complement not only in terms of receptor expression levels but also in terms of the expression of signaling effectors and modulators. Indeed, this is likely to be the explanation behind the fact that morphine can induce receptor internalization in particular sets of neurons and not others [59].

The impact of the different phosphorylation profiles on MOR function and regulation at a cellular level is now well established. As mentioned above, phosphorylation of Ser^375^ is essential but not sufficient to elicit receptor internalization. In fact, higher-order phosphorylation of the ^370^TREPHSTANT^379^ region is required for the recruitment of β-arrestin2 to drive clathrin-coated pit formation and receptor endocytosis [40,42,51]. In contrast, phosphorylation of the ^354^TSST^357^ motif does not seem to be required for receptor endocytosis. Rather, it seems to be related to the dynamics of ligand and effector binding. Birdsong and colleagues showed that phosphorylation of the ^354^TSST^357^ motif facilitates the generation of the high-affinity state of the receptor upon high efficacy agonist stimulation, suggesting that phosphorylation of such motif can have an allosteric effect on ligand binding. Such phosphorylation was proposed to be a mechanism for “imprinting” the receptor with a memory of prior activation [60,61]. More recently, it has been shown that by allosterically modulating ligand binding, this region participates in the stability of the interaction between MOR, GRK2 and β-arrestin2 as alanine mutations of this motif resulted in a dramatic change in the dynamics of recruitment of these two proteins [40].

Other intracellular kinases have been described to phosphorylate MORs [36]. Although they are not the focus of this review, the most prominent of these is Protein Kinase C (PKC). Ser^363^ and Thr^370^ have been identified as residues that undergo both morphine and phorbol 12-myristate 13-acetate (PMA)-induced phosphorylation by PKC [62,63]. However, while it is clear that PKC plays an important role in morphine-induced desensitization of MOR and morphine-induced tolerance [42,64,65], it is still unclear whether this is the result of MOR phosphorylation or the phosphorylation of other signaling effectors [40,42].

**Figure 2:**
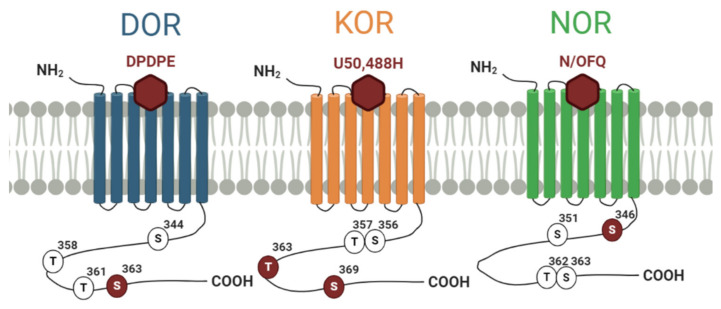
Phosphorylation sites for DOR, KOR and NOR. The primary phosphorylation sites are indicated in maroon.

Phosphorylation barcodes have also been described for the other members of the OR family (Figure 2). Of the 7 putative phosphorylation sites at the C-tail of DOR, only the 4 distal residues show prominent phosphorylation [66]. Agonist-induced, GRK2/3-dependent phosphorylation has been shown to occur at Ser^363^ and Thr^358^, with Ser^363^ acting as a primary, essential site. Thr^361^ has been shown to be important for kinase recognition and needed for appropriate Thr^358^ and Ser^363^ phosphorylation [66,67,68]. Phosphorylation of Ser^344^ by PKC has also been suggested [69] although definitive evidence of this modification is still lacking. While GRK2-mediated phosphorylation of the DOR is required for its internalization, the endocytic fate of the receptor seems to depend on the differential engagement of β-arrestin1 versus β-arrestin2 [70]. Site-directed mutagenesis and the use of phospho-specific antibodies have shown agonist-induced phosphorylation of Ser^356^, Thr^357^, Thr^363^, Ser^369^ at the C-tail of KOR, which are essential for internalization. In particular, residues Thr^363^ and Ser^369^ act as primary phosphorylation sites, and phosphorylation of either Thr^363^ or Ser^369^ is critical for phosphorylation of Ser^356^/Thr^357^ [71]. GRK2, GRK3, GRK5 and GRK6 are involved in the agonist-induced phosphorylation of all four residues (albeit to different degrees) [72], while PKC participates in both agonist-dependent and -independent phosphorylation of the receptor. Finally, phosphosite specific antibodies revealed a dynamic agonist-induced phosphorylation of NOR at 4 residues Ser^346^, Ser^351^, Thr^362^ and Ser^363^. This multisite phosphorylation is also hierarchical with Ser^346^ as primary phosphorylation site and is mediated mainly by GRK2 and GRK3 [73].

## 4. The Role of Phosphorylation on MOR Desensitization

GPCR phosphorylation is now an established mechanism underlying acute agonist-dependent desensitization, namely the rapid decline in signaling in the continued presence of agonist. In the case of the MOR, acute desensitization has been suggested to play a role in the development of antinociceptive tolerance. In this context, electrophysiological approaches performed on different cell lines and in rodent brain slices have been extensively used [74]. The most direct evidence of the contributions of GRKs to MOR desensitization is the observation that the pharmacological inhibitor of GRK2 and GRK3, compound 101, inhibits the opioid-induced desensitization of GIRK currents in rodent locus coeruleus (LC) neurons [75].

Moreover, upon the identification of the phosphorylation barcode of the MOR, in 2015 two parallel studies used mutant receptors (although not exactly the same mutations) to assess the impact of phosphorylation on MOR desensitization. To investigate the role of the STANT and TSST motifs on acute desensitization in live neurons, Birdsong et al. [61] injected AAV2 viruses encoding wild-type or mutant MOR deficient in specific phosphorylation site(s) into MOR knock-out mice and assessed receptor function using whole-cell voltage-clamp recordings in acute brain slices. Surprisingly, when viruses were injected in the medial thalamus, all groups (WT MOR or mutant MOR), showed a similar acute decline in the peak current that occurred in the continued presence of the desensitizing concentration of Met-enk. However, differences between WT and mutants were apparent when comparing a more sustained desensitization of the current evoked by a low concentration of Met-enk a few minutes after desensitization relative to that evoked before desensitization. Mutation of both motifs (TSST and STANT) resulted in a significant decrease on sustained desensitization while mutation of the STANT motif alone was not significantly different than the combined mutant, thus suggesting that this motif played a more significant role in this sustained desensitization. Such discrepancies between the different measures of desensitization were hypothesized to reflect either different processes or, alternatively, that the acute decline in signaling was a less sensitive measure of receptor function. However, these results suggesting a more significant role for the STANT motif in MOR desensitization were in contrast with those obtained by Yousuf et al. who performed perforated patch-clamp recordings in AtT20 cells [42]. In this study mutation of all 11 potential phosphorylation sites was required to eliminate the desensitization induced by Met-enk, but not morphine. In this study, the desensitization induced by morphine was only attenuated in the phosphodeficient mutant MOR upon the concomitant addition of a PKC inhibitor (see below). The discrepancy between these two studies was partially resolved upon assessment of STANT, TSST and phosphodeficient mutant MORs using both, perforated and whole-cell patch-clamp in parallel [40]. This study confirmed that mutation of the STANT motif reduced desensitization in whole-cell patch-clamp assays but not when perforated patch-clamp was employed and suggested the action of cytoplasmic mediators of receptor desensitization that would diffuse out of the cells during whole-cell but not perforated patch-clamp recording conditions. These mediators still remain to be identified.

The study of desensitization using neuronal recordings in brain slices has taken advantage of the ability to virally express phosphorylation-deficient mutant MOR receptors in knockout animals as well as the development of knockin animals that express mutant MOR in which C- terminus phosphorylation sites are replaced with alanine. Met-enk induced desensitization was largely blocked upon viral expression of a phospho-deficient MOR mutant in LC neurons of MOR knock-out rats [44]. Similarly, in LC neurons of a knock-in mouse expressing the same phospho-deficient MOR suggested that mutation Ser and Thr on the C-terminus of MOR results in a profound reduction, but not elimination, of two measures of acute desensitization; where the small residual component is likely due to heterologous desensitization [45]. These studies also showed that the development of analgesic tolerance to fentanyl in the phosphodeficient mice was significantly reduced while tolerance to morphine was reduced but not eliminated. Finally, in a more recent study, using viral injection of different MOR phospho-mutants and electrophysiological approaches to distinguish desensitization and cellular tolerance, Arttamangkul et al. have shown how all phosphorylation sites contribute, in varying degrees, to acute desensitization and long-term tolerance illustrating how these two processes are not necessarily linked [37].

It is important to note that, while GRKs are key components for MOR desensitization induced by most opioid agonists, it is also well acknowledged that other kinases contribute to MOR desensitization. As mentioned above, this is particularly the case for morphine and PKC. Indeed, there is a large body of literature using mostly electrophysiological approaches that supports the role of different PKC isoforms in both, MOR acute desensitization [40,42,76,77,78] and morphine-induced antinociceptive tolerance [79,80,81,82].

## 5. Signaling Consequences of MOR Phosphorylation

Receptor phosphorylation and the subsequent binding of arrestins has been shown to induce a second wave of signaling for some GPCRs [83,84]. In the context of the MOR, there is some evidence that suggests that arrestins are required for distinct ligand-dependent signaling events. DAMGO and morphine have been shown to elicit different spatiotemporal signaling profiles in recombinant cells and dorsal root ganglia neurons [43]. Activation of MOR by DAMGO induces cytosolic and nuclear ERK phosphorylation, while activation with morphine limits ERK activation to the cytosol [43,85]. In contrast, morphine induces a localized plasma membrane PKC activation that is not detected upon DAMGO stimulation. These effects seem to be related to the phosphorylation state of the receptor, as the mutation of Ser^375^ or all Ser/Thr residues in the C-tail of MOR, results in a change of these signaling profiles [43]. Moreover, the capacity of an opioid to activate nuclear ERK depends on its ability to induce receptor translocation within the plasma membrane prior to receptor internalization. Further, recent work suggests that this ligand-induced changes in diffusion across the plasma membrane require GRK2/3 activation, as they are impaired upon GRK2/3 inhibition, and may involve a dimerization step that precedes internalization [43,86,87].

Biased agonism is a pharmacological paradigm that describes the ability of different ligands, binding to the same receptor to stabilize distinct conformations of that receptor that differentially engage with a distinct signaling pathways to the relative exclusion of others. At the MOR, efforts to identify biased ligands were fueled by observations that in mice lacking β-arrestin2, morphine analgesia was enhanced and prolonged while tolerance, respiratory depression and constipation were diminished [88,89] suggesting that while the analgesic effects of opioids are β-arrestin-independent, β-arrestin2 is responsible for the adverse effects of these drugs. This arrestin hypothesis has dominated MOR drug discovery over the last decades and numerous reports exist of opioid agonists proposed as G protein biased ligands with a relatively lower propensity to stimulate β-arrestin recruitment (and MOR phosphorylation). Among those proposed biased ligands, and the first to be discovered, is oliceridine, which has recently been approved by the FDA for the management of acute severe pain in clinical settings. However, recent evidence has challenged the original finding in the β-arrestin2 knock-out mice [45,90,91] and alternative pharmacological explanations have been put forward to explain how low efficacy ligands, such as most of the proposed biased agonists, can induce robust G protein signals and limited β-arrestin recruitment and result in improved therapeutic profiles [31,92,93,94]. Demonstrating such alternative mechanisms in vivo represents one of the most pressing challenges in opioid pharmacology.

## 6. In Vivo Relevance of MOR Phosphorylation

The use of genetically modified mice has provided key information on the mechanisms that mediate the physiological effects of opioids, including analgesia, tolerance, reward, dependence, withdrawal, respiratory depression and constipation. Insights into the physiological roles of MOR phosphorylation have been provided by assessing the actions of opioids in knock-in mice expressing phosphorylation-impaired MORs or knock-out mice lacking the expression of a particular GRK or β-arrestin isoform.

### 6.1. β-arrestin Knock-Out Mice

Key early studies in β-arrestin2 knock out mice have impacted the field of opioid research for decades. These studies showed how in mice lacking β-arrestin2 (but not β-arrestin1), morphine analgesia was enhanced and prolonged, antinociceptive tolerance was diminished, reward was enhanced and dependence was unchanged [88,95,96]. Later, these same mice were shown to have reduced morphine-induced respiratory depression and acute constipation [89]. Altogether these findings spearheaded the field of biased agonism at the MOR as they provided physiological evidence of two potentially distinct signaling pathways mediating the analgesic versus the adverse (β-arrestin-mediated) effects of opioid drugs. However, recent studies suggest that the original β-arrestin2 knock-out studies may have been confounded by the genetic background of the mice, rendering them less sensitive to the respiratory effects of morphine [90] and therefore challenging the β-arrestin2 hypothesis of opioid-induced side effects.

### 6.2. Knock-In Mice Expressing Mutant MORs

In knock-in mice expressing the phosphorylation-deficient S375A mutant of the MOR, morphine and fentanyl exhibited greater dose-dependent antinociceptive responses than in the corresponding wild-type mice [46]. This is in line with the idea of Ser^375^ phosphorylation being involved in acute MOR desensitization. Interestingly, these mice had reduced antinociceptive tolerance to high efficacy agonists such as DAMGO or etonitazene, but not to morphine, again supporting the hypothesis that the development of antinociceptive tolerance occurs via different mechanisms depending on opioid agonist efficacy. More recently, knock-in mice expressing a phospho-deficient MOR (11ST/A) has been generated [45]. As mentioned above, acute desensitization of MOR is severely impaired in LC neurons of these mice compared to WT. In line with this lack of desensitization, the analgesic effects of fentanyl and morphine are enhanced and prolonged. However, these mice show exacerbated opioid-induced respiratory depression and constipation and in Kölliker fuse neurons of these mice, opioids still induce GIRK currents. These results are at odds with the β-arrestin-dependent, G-protein-independent signaling mechanisms previously proposed to mediate opioid-induced side effects. While reward and physical dependence signs were still retained in these mice, the antinociceptive tolerance was completely abrogated (fentanyl) or significantly reduced (morphine).

### 6.3. GRK Knock-Out Mice

The effects of opioids in mice lacking specific GRK isoforms have also been studied, however these are mostly limited to morphine’s actions.

- *GRK2*. Despite GRK2 being a key kinase for the regulation of MOR, studying the role of this kinase in vivo is still a challenge. Mice with a homozygous deletion of GRK2 are not viable, and heterozygous GRK2+/- mice show similar antinociceptive responses to morphine than the corresponding WTs. Use of conditional or inducible GRK2 knock-out animals or in vivo administration pharmacological inhibitors represent future strategies to address this.
- *GRK3*. Studies with mice lacking GRK3 suggest that this kinase plays a significant role in the development of tolerance to fentanyl both in vitro in mammalian brain slices and in the in vivo analgesic response [47]. Indeed, in vivo phosphorylation of Thr^370^, Ser^375^ and Thr^379^ by etonitazene and fentanyl was predominantly mediated by GRK3 [48]. In contrast, morphine-induced antinociceptive tolerance was not affected by this deletion. These data are in line with the in vitro results discussed above that suggest that both GRK2 and GRK3 are the main kinases phosphorylating MOR upon activation with high efficacy agonists but not low efficacy agonists such as morphine.
- *GRK5*. In vivo, morphine has been shown to promote selective phosphorylation of Ser^375^ which is predominantly mediated by GRK5 [48]. GRK5 knockout mice exhibited reduced antinociceptive responses after morphine administration and developed morphine tolerance similar to wild-type mice but fewer signs of physical dependence. Different to what had been previously observed in the Ser375A knock-in mouse, the rewarding effects of morphine were diminished in GRK5 knockout mice, suggesting that modulation of this kinase could represent a potential new approach for preventing opioid addiction while maintaining their analgesic properties.
- *GRK6*. Upon ablation of the GRK6 gene, morphine has been shown to induce greater locomotor activity and less constipation while not affecting morphine-induced thermal antinociception, analgesic tolerance and physical dependence [49]. These results suggest a role of GRK6 in regulating some, but not all, morphine-mediated responses. They also highlight the relevance of understanding the protein complement of cells in different opioid receptor-expressing tissues.

### 6.4. PKC

There is strong ex vivo electrophysiological evidence of the importance of PKC for morphine-induced desensitization [36]. Moreover, the early studies in β-arrestin2 KO mice suggested the presence of a parallel PKC-dependent mechanism mediating morphine antinociceptive tolerance [97]. Despite this, in vivo studies on the role of the different PKC isoforms in opioid-induced behaviours mainly rely on the use of pharmacological inhibitors with mixed isoform activity and are thus still difficult to interpret.

## 7. Conclusions

GRKs are major regulators of opioid receptor function. The distinct phosphorylation profiles elicited by different opioid agonists are highly dependent on their intrinsic efficacy, tightly regulating plasma membrane reorganization of receptors, desensitization and internalization. In vivo, the importance of MOR phosphorylation for the chronic effects of opioids is undisputed. However, the cell-type dependent mechanisms that are controlled by this phosphorylation are just starting to be unraveled. Future directions in this area will rely on genetic approaches that allow the selective knock-down of specific kinases in particular cells or tissues. Such understanding will help the development of opioid ligands with the optimal pharmacological properties that allow for the provision of pain-relief with minimal (or absent) acute and chronic side-effects.

## Figures and Tables

**Table 1:** Genetic and pharmacological approaches used to assess the roles of GRKs in MOR function.

	Approach	Effects on MOR Function	References
**Kinase inhibitors**	GRK2/3 (compound 101)—stabilizer of GRK2/3 in closed, non-catalytic conformation [38]	Reduced desensitization	[39,40]
		Reduced MOR phosphorylation	[31]
		Reduced arrestin recruitment	[40]
	GRK2 inhibitor (CCG258747)-	Reduced MOR internalization	[41]
**Mutation of C-tail Ser/Thr**	Cell lines	Reduced desensitization	[40,42]
		Reduced GRK2 and arrestin recruitment	[40]
		Changes in MOR signaling	[43]
	Neurons and brain slices	Reduced desensitization	[44]
		Impact on long-term tolerance	[37,45]
	Knock-in mice	Increased antinociceptive responses	[46]
		Reduced desensitization	[45]
**Kinase knock-out mice**	GRK3	Reduced antinociceptive tolerance	[47] [48]
	GRK5	Reduced antinociceptive response Loss of morphine reward Reduced morphine tolerance	[48]
	GRK6	Greater locomotor activation Reduced constipation	[49]
